# Rethinking collaboration: developing a learning platform to address under-five mortality in Mpumalanga province, South Africa

**DOI:** 10.1093/heapol/czz047

**Published:** 2019-06-26

**Authors:** Lucia D’Ambruoso, Maria van der Merwe, Oghenebrume Wariri, Peter Byass, Gerhard Goosen, Kathleen Kahn, Sparara Masinga, Victoria Mokoena, Barry Spies, Stephen Tollman, Sophie Witter, Rhian Twine

**Affiliations:** 1 Centre for Global Development and Institute of Applied Health Sciences, University of Aberdeen, Aberdeen, UK; 2 Department of Public Health and Clinical Medicine, Umeå Centre for Global Health Research, Epidemiology and Global Health, Umeå University, Umeå, Sweden; 3 MRC/Wits Rural Public Health and Health Transitions Research Unit (Agincourt), School of Public Health, University of the Witwatersrand, Johannesburg, Johannesburg, South Africa; 4 Medical Research Council (MRC) Unit, The Gambia at London School of Hygiene and Tropical Medicine (LSHTM), London, UK; 5 Mpumalanga Department of Health, Nelspruit, South Africa; 6 INDEPTH Network, Accra, Ghana; 7 Institute for Global Health and Development, Queen Margaret University, Edinburgh, UK

**Keywords:** health policy and systems research, under-five mortality, South Africa, verbal autopsy, participatory action research

## Abstract

Following 50 years of apartheid, South Africa introduced visionary health policy committing to the right to health as part of a primary health care (PHC) approach. Implementation is seriously challenged, however, in an often-dysfunctional health system with scarce resources and a complex burden of avoidable mortality persists. Our aim was to develop a process generating evidence of practical relevance on implementation processes among people excluded from access to health systems. Informed by health policy and systems research, we developed a collaborative learning platform in which we worked as co-researchers with health authorities in a rural province. This article reports on the process and insights brought by health systems stakeholders. Evidence gaps on under-five mortality were identified with a provincial Directorate after which we collected quantitative and qualitative data. We applied verbal autopsy to quantify levels, causes and circumstances of deaths and participatory action research to gain community perspectives on the problem and priorities for action. We then re-convened health systems stakeholders to analyse and interpret these data through which several systems issues were identified as contributory to under-five deaths: staff availability and performance; service organization and infrastructure; multiple parallel initiatives; and capacity to address social determinants. Recommendations were developed ranging from immediate low- and no-cost re-organization of services to those where responses from higher levels of the system or outside were required. The process was viewed as acceptable and relevant for an overburdened system operating ‘in the dark’ in the absence of local data. Institutional infrastructure for evidence-based decision-making does not exist in many health systems. We developed a process connecting research evidence on rural health priorities with the means for action and enabled new partnerships between communities, authorities and researchers. Further development is planned to understand potential in deliberative processes for rural PHC.

**Key Messages**

- Despite progressive health policy, implementation challenges limit progress in reducing health inequalities in rural South Africa. The introduction of a ‘learning platform’ connecting stakeholders from provincial and district health systems with researchers can promote co-production and use of research evidence to inform policy and programmes.
- Structured spaces for dialogue and deliberation in local policy and planning can extend the potential of research to inform service organization and delivery. Sustaining such platforms via integration into routine activities, including key stakeholders within and beyond the health system, and shared planning, design, learning and adaption are key to realizing potential.

## Introduction

Fifty years of apartheid in South Africa resulted in entrenched racial inequalities (Smith, 2005). Following years of organized local and international pressure, South Africa transitioned to a democratic state in 1994, making constitutional commitments to the right to health as part of an inclusive, pro-poor primary health care (PHC) approach. Despite the ‘unhurried, strategic vision’ (Mehl *et al.*, 2018) of the South African Department of Health (DoH), implementation is beset with challenges in a public health system characterized by decades of chronic underinvestment, human resource crises, corruption, poor stewardship and deteriorating infrastructure (Coovadia *et al.*, 2009; McIntyre, 2012) and disproportionate burdens of avoidable mortality persist with widening inequalities documented recently (Bradshaw *et al.*, 2003; Harris *et al.*, 2011; Gómez-Olivé *et al.*, 2014; Mayosi and Benatar, 2014; World Bank, 2018a). In this scenario, there is an urgent need for evidence on the processes through which policy is implemented for people who are socially excluded from access to health systems. Evidence alone is insufficient to enable change, however. Evidence needs to be understood, owned and used to effectively inform planning and practice. Despite considerable interest in evidence-based policy over the past three decades, there is comparatively less attention to the processes through which evidence is produced and used in policy-making (Parkhurst, 2017).

Health policy and systems research (HPSR) has emerged recently as an approach to embed research more completely in policy-making and implementation, encouraging active engagement between researchers and decision-makers (WHO, 2012; Hafner and Shiffman, 2013; de Savigny *et al.*, 2017; Cleary *et al.*, 2018a). HPSR is an interdisciplinary and problem-oriented, prioritizing research conducted with and for policy stakeholders negotiating solutions to health systems issues and establishing a nexus between providers and socially excluded groups (Gilson *et al.*, 2011; Sheikh *et al.*, 2011; Gilson, 2012). In this sense, ‘embeddedness’ is a hallmark of rigour in HPSR (Walley *et al.*, 2018; WHO AHPSR, 2018). There is normative support around the emerging HPSR paradigm. The Sustainable Development Goals call for equitable and strong institutions with a focus on governance, complexity and interconnectedness, and Universal Health Coverage raises ongoing needs to understand how to deliver accessible, affordable and accountable services (WHO, 2017; *Scott et al.*, 2018). As a result, consensus is developing around methods and for HPSR as a core function in every health system (de Savigny *et al.*, 2009; Gilson, 2012; WHO, 2012; Erasmus *et al.*, 2014; Akhnif *et al.*, 2018).

Our aims were to develop a collaborative learning platform in which we worked as co-researchers with health authorities in a rural province to generate evidence of practical relevance on implementation processes with and for people excluded from access to health systems. The objectives were to engage health systems stakeholders to identify research priorities, design research, interpret data, identify remedial actions and reflect on the process. This article reports on insights brought to the process by systems stakeholders and considers application more generally.

## Methods

### Study setting

We worked in Mpumalanga province in rural northeast South Africa. Mpumalanga is one of nine provinces in the country, with a population of 4.4 million (7.8% of the national population). More than half the population is rural (nationally it is around one-third) (SSA, 2017b; World Bank, 2018b). In 2014, reflective of the situation nationally, unemployment in the province was 26%, with 51% living in poverty (SSA, 2017a). In 2015, life expectancy was 50 and 53 years for males and females, respectively, lower than the national average of 60 and 67 years, and under-five mortality was 41 deaths per 1000 live births in 2012, which is comparable nationally (MDoH, 2015; Bamford *et al.*, 2018; WHO, 2019). Mpumalanga Department of Health (MDoH) delivers need-based services through an integrated health system covering three districts and 18 local municipalities (MDoH, 2015). The MDoH structure includes five strategic directorates: HIV/AIDS, sexually transmitted infections and TB Control (HAST); communicable disease control; non-communicable diseases (NCD); maternal, child, women and youth health and nutrition (MCWYH&N); and research and epidemiology. The research was located at the MRC/Wits Rural Public Health and Health Transitions Research Unit, which hosts the Agincourt Health and Socio-Demographic Surveillance System (HDSS). Established in 1992, Agincourt is the oldest HDSS in South Africa, collecting longitudinal data on vital events for a population of approximately 116 000 (Kahn *et al.*, 2012).

### Engaging the health authority to identify evidence gaps

The first step was to identify evidence needs with the health authorities. We initiated the partnership with MDoH through the Agincourt HDSS Stakeholder Engagement Office, a group with long-term links to different levels and sections of the health system. In 2013 we arranged an initial engagement with the MCWYH&N Directorate in which the aims, scope and HPSR approach were introduced, and a process was proposed to work as co-researchers to produce and analyse data of practical relevance. In the initial engagement, MDoH articulated a lack of timely and robust evidence on contributory circumstances and events occurring outside facilities in under-five deaths as a priority area on which the Directorate had little information. On this basis, we collected data on under-five deaths in rural villages in the Agincourt HDSS as follows:

### Defining size and scope of the problem (verbal autopsy)

We acquired verbal autopsy (VA) data from routine surveillance in Agincourt HDSS to quantify levels and causes of under-five deaths in the site. VA is a pragmatic approach to ascertaining medical causes of death in populations where registration systems are incomplete or absent (Nichols *et al.*, 2018). Responding to the evidence gaps identified with MDoH, we further developed prior modifications to the VA method to capture additional information on circumstances and events outside facilities at the time of death. Data were acquired on all 54 under-five deaths identified and investigated with VA in Agincourt HDSS over a 2-year surveillance period 2012–13. These data were processed using InterVA-4, a public-domain probabilistic model for VA data interpretation (www.interva.net) to generate cause-specific mortality fractions (Byass *et al.*, 2012). The tool processes VA input indicators as defined in World Health Organization (WHO) VA standard and delivers WHO-defined cause of death categories compatible with the International Classification of Diseases version 10 (ICD-10). The VA data revealed high levels of under-five mortality owing to infections (>70%) and multiple and reinforcing barriers to access reflected in not calling for help and not travelling to a facility at the time of death. The analysis is reported elsewhere (D’Ambruoso *et al.*, 2016).

### Exploring problems from community perspectives (participatory action research)

We introduced a participatory action research (PAR) process in the Agincourt HDSS study area in 2015 to elicit local knowledge on under-five mortality and priorities for action. Participatory methodologies generate local knowledge on the relationships between social conditions and health for action and learning on action (Loewenson *et al.*, 2014). Service users and providers at village level were convened and consulted on under-five deaths, with experiences and perspectives elicited and systematized using methods such as ranking, diagramming and participatory photography (Catalani and Minkler, 2010). Community stakeholders identified social and structural root causes of under-five mortality as inadequate housing, high unemployment and lack of clean, safe water, and perceptions of poor quality of care in clinics related to long waiting times, lack of triage, overcrowding, delays in treatment, medication shortages and confidentiality breaches. Community stakeholders also developed priorities for action to reduce unemployment, provide clean water, expand community health education, and clinics with accountable and responsive staff. This analysis is also reported separately (Wariri *et al.*, 2017).

### Collaborative formulation of solutions

The final stage was to take the VA and PAR data back to health systems stakeholders to analyse, interpret, formulate recommendations for policy, planning and practice and reflect on the process. Following initial organization and analysis, we re-convened MDoH stakeholders. With steer from the MCWYH&N directorate, stakeholders were identified to include a range of levels and perspectives. Two formal workshops were held from May to November 2016: one with the provincial directorate for MCWYH&N and one with provincial directorates and district departments (MCWYH&N, NCDs, PHC, and research and epidemiology). Both workshops were held in the City of Mbombela as a convenient location for participants. In the workshops, investigators and stakeholders worked together as a plenary group to analyse and interpret the VA and PAR data with reference to local policy and planning, and reflected on the utility of the process. We collected data in presentations, registers, minutes, observational notes and reflective journal data to record interpretations and proposed responses. The analysis was further developed through informal exchanges in person and in writing. In the following section, we present the provincial and district stakeholders’ substantive interpretations and recommendations to illustrate the explanatory potential of the collaborative learning approach.

### Ethical considerations

For the VA component, secondary analysis of existing anonymized data was undertaken and approved by the authors’ institutes. For the PAR, informed consent was sought from all participants, all identifiable data were anonymized, approvals obtained from the authors’ institutes and from the provincial health authority. The provincial and district health systems stakeholders’ interpretations are presented as a professional working group in this jointly authored piece.

## Results

**Figure 1:**
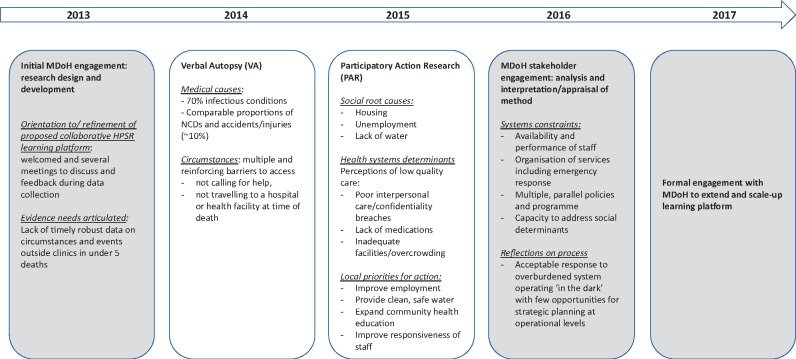
Schema of data gathering phases (formal MDoH engagements in shaded boxes, informal engagements represented by arrow).

Detailed interpretations of the VA and PAR data were developed with officials from MDoH, with several systems deficiencies identified as contributory to under-five deaths and a series of corresponding recommendations was developed. These are presented below and in Table 1. A summary of the data gathering phases is provided in Figure 1.

**Table 1:** Systems constraints and recommendations (arranged according to feasibility of implementation)

Systems constraints	Recommendations		
	Immediate term	Medium term	Long term
1: Improve availability and performance of health workers	Promote improved interpersonal behaviours and everyday practices with special attention to ensuring respect and maintaining patient confidentiality.	Improve basic data on staff numbers in rural PHC clinics; Develop staff-led quality improvement linked to higher levels of the health system to identify and address staffing and performance issues.	Advocate for increased staff numbers in rural PHC clinics.
2: Strengthen service organization, infrastructure and emergency response	Develop flexible clinic appointments in clinics where patients are seen in blocked intervals rather than at specific times.	Improve organization of ambulance services using GPS to verify and prioritize calls and with dedicated emergency and functional rapid response systems; Improve monitoring via GPS to strengthen existing tracking and EMS responsiveness and to inform local EMS planning.	Engage with higher levels of the system and beyond MDoH in priority setting and capital investment to improve consulting rooms and other infrastructure.
3: Destabilising effects of multiple parallel policy initiatives		Promote local data cultures to address multiple top-down policy and programming changes and parallel initiatives; Improve local level autonomy and capacity to shape policy initiatives.	
4: Improve capacity to address social determinants of under-five deaths	Engage CHWs to design and deliver health promotion, health education and community-based care specific to local needs; Promote use of information and digital technologies for two-way communication between providers and patients.	Develop co-ordinated community health responses consolidating WBOTs, CHWs and HBCs operating through NGO/NPOs foster inclusion of CHWs in improved data and data processes.	Extend collaborative work to departments of labour, housing, social development, water and sanitation.

EMS, emergency medical services.

### Health worker availability and performance

Insufficient health workers were identified as a primary contributory factor in under-five deaths. It was noted that all forms of health workforce mal-distribution—public/private, rural/urban and primary/tertiary—affect PHC in Mpumalanga. Basic staff numbers are also difficult to ascertain, which further constrains planning. Additional challenges were identified in terms of skills, competencies, support and motivation. Combined with the staffing issues, these were seen to collectively limit the quality of care that frontline workers can reasonably provide, fostering systemic attitudinal and motivational problems. In response, changes to everyday behaviours in clinics, particularly around respectful care and confidentiality, were recommended as an immediate low/no-cost intervention capable of improving interpersonal aspects of care, which was identified in the PAR as major problem. It was recognized that while behaviour change may be neither feasible nor sustainable in the absence of attention to staffing issues, simple changes to everyday practices were viewed as having the potential to drive change responding to local concerns. In the medium term, appreciative staff-led quality improvement was recommended to support broader changes in clinic cultures for improved competencies, supervision and monitoring staff levels. It was acknowledged that while staff increases are unrealistic immediately, better local data linked to higher levels of the health system may be a means to progress towards longer-term goals.

### Service organization and infrastructure

Inadequate service organization and infrastructure were identified as further contributory factors. Overcrowding was a specific issue discussed in detail. Health systems stakeholders provided important insights into how the practice of holding clinics in the mornings leads to flooded clinics at certain times of day, exacerbating staffing problems and causing long waiting times and delayed or postponed consultations. Flexible clinic appointments (where patients are seen within blocked intervals rather than at specific times) combined with improved recording and regular review were proposed as a no cost/low-cost measure to reduce overcrowding. Flexible appointments were also seen as able to address transport and other indirect costs, identified as problematic in the VA data, by reducing the chances of patients being turned away.

The lack of consulting rooms was a further contributory factor identified. Participants described how many clinic buildings date back to when the area was a homeland and significantly smaller populations were provided with limited and poor quality services. Public facilities now cater for much larger populations and are competing for infrastructure budget with other projects, resulting in higher throughput than is manageable, maintenance failures and a lack of maintenance planning. It was also recognized that facility maintenance is not within the control of MDoH and involves the provincial Department of Public Works, Roads and Transport (DPWRT). Actions to improve consulting rooms and other infrastructure were therefore seen to require priority setting and capital investment at higher levels of the system and beyond MDoH, with whom engagement would be necessary.

The time-critical nature of under-five deaths was also discussed. While MDoH reported 70% of responses <40 min in rural areas in 2016/17, incomplete monitoring and shortages of personnel and ambulances are key challenges (MDoH, 2017). Improved organization of ambulance services was recommended with Global Positioning Systems (GPS) to verify and prioritize calls. While many vehicles have tracking systems for theft and radios, the systems do not allow real-time tracking and radios operate only within the repeater range with tracking lost intermittently. Broader use of GPS was recommended as a medium-term approach to strengthen emergency responsiveness by enabling the locations of ambulances to be known at all times and in real time. Electronic logging of response times was also seen as a way of generating data to inform planning. While costs were acknowledged, the potential to be offset by efficiency gains was noted.

### Multiple policy/programme changes and parallel initiatives

Multiple top-down policy and programming changes and parallel initiatives were identified as a further systems constraint. It was noted that well-intentioned policies, programmes, audits and other initiatives are dictated from higher levels and lack local relevance. Many parallel initiatives were described as having an overall destabilising effect among over-burdened health workers, compounding problems in an already constrained system. As a result, objectives are often not achieved and results manipulated to show progress. This was described as ‘changing numbers rather than really affecting change’. The situation was seen to negatively affect programmes and audits, overlooking local innovations and capabilities. Local level autonomy and capacity to shape policy was emphasized to address the destabilizing effects of multiple top-down policies and initiatives. Lower level input to policy formulation and implementation was considered pivotal to resolving poor policy co-ordination and its consequences for quality, accessible care for under-fives. This recommendation confirmed and consolidated the prior recommendations on improving staffing, service organization and infrastructure through local data and data processes.

### Social determinants of under-five deaths

The final contributory factor identified was the intense pressures placed on the health system by social and structural drivers of under-five mortality. These were identified in the VA and PAR data to include: widespread poverty, unemployment, inadequate housing, lack of water and pervasive financial barriers. There was surprise among stakeholders regarding conditions of poverty in rural villages, revealed in the visual PAR data, and the lack of connectivity between the authorities and social conditions of underserved populations was recognized. Extending the platform to include government departments external to health (including labour, housing, social development, water and sanitation) was recommended to improve capacity to address entrenched drivers of under-five deaths. The earlier interpretations had converged around the need for local evidence systems within and between levels within DoH. Here, we considered extending the learning platform outside MDoH to advance multi-sectoral action between departments. Consolidating community health initiatives was also recommended as a medium-term response to the social determinants of under-five deaths. While a considerable lay health workforce exists [including community health workers (CHWs), home-based carers (HBC) operating through non-governmental and non-profit organizations (NGO/NPOs), lay counsellors and direct observed treatment supporters] it is fragmented and under-resourced. Consolidation of CHWs through inclusion in local data and extending health promotion, health education and community-based care was recommended, as was the use of information and digital technologies for two-way communication between providers and patients.

### Summary

Considered as a whole, the recommendations ranged from immediate low- and no-cost reorganization of existing services to those where responses from higher levels and/or outside the health system were required. Arranging the findings according to feasibility of implementation provided an integrated set of recommendations (Table 1). This, in turn, conferred a sense of positivity and control. Modest changes were seen to be achievable immediately, and that through the shorter-term actions, progress towards medium and longer-term agendas could be facilitated. In the following section, we discuss the generalisability of these findings to showcase the analytical potential of the process. This is followed by a critical appraisal of the acceptability and reproducibility of the process.

## Discussion

### Informing policy, planning and practice

A series of system-wide deficiencies were identified as contributory factors in under-five deaths and detailed recommendations were developed related to formal and informal spheres of action, influence and change identifying potential leverage points to influence systems performance. It is unlikely that these insights would have been gained with researcher input alone, i.e. without the HPSR-informed approach. The findings are consistent with, and add to, previous research and government initiatives from the region (Table 2). Common to the interpretations was the need for local data and the participation of providers and managers in the planning process. It was emphasized that health workers and managers have substantial knowledge of how health policy is implemented through daily decisions about eligibility and access in resource-scarce, demand-intensive environments, but their voices are often overlooked in policy, planning and management. Research from elsewhere in South Africa identifies punitive ‘compliance cultures’ in sub-district leadership, whereby staff passively follow rules and standards, overlooking the transformative potential of ‘bottom up creativity and innovation’ (Cleary *et al.*, 2018a; Gilson *et al.*, 2018).

**Table 2:** Health systems stakeholders’ analysis, consistencies with and additions to policy and research literature

Systems constraints	Recommendation	Support in policy and academic literature	Contributions to policy and academic literature
Health worker availability and performance	Behaviour change to improve respect and confidentiality	Research elsewhere in South Africa has identified that: ‘caring, respectful communication, individual acts of kindness, and institutional flexibility and leadership may mitigate key access barriers and limit threats…fostering more positive forms of inclusion and facilitating easier access’ (Harris *et al.*, 2014).	In Mpumalanga and the Agincourt area specifically, a focus on maintaining patient confidentiality may be a route to facilitate access and improve widely held perceptions of poor quality of care in clinics.
	Staff led quality improvement	Research in the Eastern Cape associated improved maternal health outcomes with supportive and visible leadership and frequent staff meetings prioritizing co-ordination and communication (Mathole *et al.*, 2018).	Application of appreciative staff-led quality improvement generally (i.e. beyond a speciality/condition-specific focus) may foster changes in clinic cultures supporting competencies and supervision and basic monitoring of staff levels.
	Increased staff numbers	Successive national plans address workforce development and planning with initiatives on affirmative student recruitment, financial incentives, foreign recruitment and compulsory service as well as commitments to strengthen the public health workforce through National Health Insurance (NHI) and the National Development Plan (NDP) (DOH, 2010, 2014, 2015a; NPC, 2012; van Rensburg, 2014). Minister of Health, Aaron Motsoaledi, has acknowledged that the country needs to triple the number of doctors to ensure success in NHI, however. The absence of systematic evaluations of human resource policy is also acknowledged as a key issue (Baleta, 2012; DOH, 2011a; Passchier, 2017).	Better local data, when sustained and linked to higher levels of the health system, may provide means to develop a solid foundation and shared basis from which to achieve longer-term goals around improving staffing levels.
Service organization, infrastructure and emergency response	Flexible clinics	The Ideal Clinic^a^ is a national quality of care framework which sets targets including to reduce waiting times in public facilities to a maximum of 3 h using measures such as flexible appointments and provides guidance on community engagement for appointment management and booking systems (Fryatt and Hunter, 2015; DOH, 2016, 2018a). The National Policy on Management of Patient Waiting Time in Out Patient Departments also promotes innovations in mobile technologies, automated patient reminders in SMS, WhatsApp messaging, e-mail and reminder calls. Although the policy recognizes the need for routine monitoring for planning purposes, challenges in effective local monitoring are acknowledged: ‘reliable data on patient waiting time is scanty and impedes efforts to find solutions for long patient waiting time’ (DOH, 2015b).	Flexible clinic appointments may address overcrowding and unaffordable indirect costs of care for repeated journeys to clinics for appointments. Regular review via processes outlined above will help address the lack of reliable data on patient waiting times, allowing innovations to be monitored, understood and modified where necessary.
	GPS in ambulances	The recommendation is consistent with national strategies for accurate triage and rapid vehicle deployment as part of efforts to operationalize more vehicles, improve communication between dispatch and EMS personnel, expand the emergency workforce and strengthen training (MacFarlane, Loggerenberg and Kloeck, 2005; Noordam *et al.*, 2015; Stein *et al.*, 2015; Anest *et al.*, 2016; DOH, 2018a).	Use of GPS to improve EMS co-ordination and response, and specifically electronic logging of response times, may provide a means to generate data to inform local planning and improvement initiatives for EMS.
	Lack of consulting rooms	As part of the implementation of National Health Insurance (NHI), in 2014 the government committed to build a minimum of 213 new clinics and 43 new hospitals, and to refurbish and re-equip 870 clinics in the 11 pilot districts of NHI (SAGNA, 2014). MDoH in the 2018/19 Annual Performance Plan (APP) (the official guiding document for health service delivery, budget, targets, etc. in the province) has an allocation to ‘build, upgrade, renovate, rehabilitate and maintain health facilities’. The 2018/19 MDoH APP also refers to ‘long-term infrastructure and other capital plans’ in addition to the current financial year plans and makes reference to conditional grants, of which one is the ‘national health facility revitalization grant’ (MDoH, 2018.)	In this setting, advancing action requires collaboration with, and inputs from, departments adjacent to health, namely the Department of Public Works, Roads and Transport (DPWRT).
Multiple parallel policy initiatives	Promote local data cultures	Research elsewhere in South Africa has identified punitive sub-district leadership, whereby staff passively follow rules and standards reinforcing and maintaining ‘compliance cultures’ in local service delivery, which overlooks the transformative potential of ‘bottom up creativity and innovation’ (Cleary *et al.*, 2018a; Gilson *et al.*, 2018).	In this application of a collaborative HPSR-informed learning platform, there is willingness and capacity to work to co-produce research evidence on priorities reflecting the perspectives of communities and local health authorities. Our process provides a means to actively engage communities in this process.
Social determinants	Engage CHWs, mHealth	The benefits of mHealth are widely documented in low- and middle-income countries (Free *et al.*, 2013; Noordam *et al.*, 2015). An important initiative in South Africa is ‘MomConnect’, an interactive platform introduced in 2014 to register all pregnant women, communicate twice-weekly health promotion information on pregnancy and create channels for feedback to connect women to services. The initiative enrolled over 500 000 women in its first year, over 56 000 in Mpumalanga, and currently reaches over 1.5 million (Pillay, 2015; Seebregts *et al.*, 2016; Barron *et al.*, 2018). Successes are attributed to sustained leadership and enabling environments including partnerships with telecommunications organizations. (Bateman, 2014; Barron *et al.*, 2018) The platform has recently been extended to NurseConnect and initiatives such as B-Wise, to help young South Africans seeking health advice online (DOH, 2018b; DOH, She Conquers, 2018).	Enabling lay health workers to employ mobile technologies to improve community health education is a logical way forward building on mHealth successes.
	Consolidate and enable CHW workforce	The national PHC Re-engineering initiative seeks to standardize CHW portfolios and integrate them into the national system in ward-based outreach teams (WBOTs^b^) as a key element of decentralizing PHC to community level (DOH, 2011b; SOPH UWC, 2012). While CHWs provide critical support in rural PHC, several systems and managerial challenges exist with stability, recognition and in relationships with professionals (Schneider *et al.*, 2008). A systems approach has been advocated to strengthen and resource CHWs with: ‘planning and regulatory systems; education, selection and training systems; supervision and psycho-social support systems; referral systems; and strengthening the related health workforce’ (Baker *et al.*, 2007).	Involvement of CHWs in learning, team approaches within the health system, as well as processes engaging in different levels, with departments adjacent to health and with local communities, offers opportunities to enable a systems approach to bringing CHWs into the national service.
	Multi-sectoral action	There is widespread recognition of the importance of multi-sectoral action to address the social and structural drivers of avoidable mortality in South Africa. Specific to children under-five are: (1) Early Childhood Development with areas of responsibility allocated to different Departments: Health, Education and Social Development; (2) the Child Support Grant administered by the South African Social Security Agency (SASSA), a parastatal linked to Department of Social Development (DSD); (3) collaboration between Department of Home Affairs, DSD and DoH in the management of children with malnutrition—e.g. additional ‘social relief of distress’ grant, administered by SASSA, through referral by DSD and ‘diagnosis’ by DoH; (d) the Child Protection Committee, a provincial, district and local structure, where issues of child safety and child protection are addressed in a multi-sectoral approach including the above stakeholders and SA Police Service, Immigration Officials and NGOs; (e) Office on the Rights of the Child, under the Premier, which has moved to DSD to oversee matters of child protection. While there is widespread recognition, effective multi-sectoral responses are lacking. HIV mainstreaming is a key example: unintentional promotion of silos working, ambiguity in the roles of other sectors, poor communication, co-ordination and power asymmetries have been identified. Authors suggest measures including explicit attention to ‘the how’ of multi-sectoral action: to the process of co-ordination within and between departments and synchronized reporting systems for tracking outcomes (DOH, 2017; Mahlangu *et al.*, 2018).	Recognition of the need for multi-sectoral action is widespread. In this application, focussed on drivers of under-five mortality outside clinics, we identified a need to collaborate with Departments adjacent to health, namely the Labour, Housing, Social Development, Water and Sanitation. Our findings suggest co-ordination and collaboration within and between departments could be supported as a long-term initiative for shared action on shared priorities. Despite being intuitively valuable, more information is needed on the specific practicalities of how to achieve effective multi-sectoral action.

EMS, emergency medical services.

a An Ideal Clinic is defined as one with adequate infrastructure (physical conditions, spaces, equipment and information and communication), staff, medicine and supplies, and administration (DOH, 2018a).

b WBOTs are designed to be nurse-led, with family health practitioners and four to five CHWs responsible for approximately 35 000 people as the main mechanism through which CHWs are trained, supported and supervised (DOH, 2011b).

Engaging practitioners in policy development and evaluation may therefore have the potential to employ these realities for positive change. Collaborative learning initiatives based on mutual trust, inclusion and respect connected to higher levels of the system and community stakeholders may help to address disconnects between policy and implementation offering solutions to local health priorities. Within and outside South Africa, research increasingly suggests that enhancing organizational and workplace cultures with more attention to *how care is delivered* can yield advances (Braithwaite, 2018; Cleary *et al.*, 2018a; Mathole *et al.*, 2018). As stated by Allwood *et al.* (2018): ‘the front line can see clearly every day what needs to change’ and ‘change is accepted when people are involved in the decisions and activities that affect them, but they resist when change is imposed by others’.

### Acceptability and utility of the process

We reflected on the process during the workshops and afterwards to develop practice and further application (Table 3). The importance of local learning initiatives to co-produce research evidence for practical application was clearly observed. The process was seen as acceptable and useful for an overburdened public system operating ‘in the dark’ in the absence of local evidence. The HPSR paradigm was welcomed given the lack of clarity among practitioners, planners and managers about realities ‘on the ground’ as well as few opportunities for strategic planning at provincial and district levels. It was also seen as a suitable approach to develop co-operation within and between authorities and communities by providing neutral spaces in which to develop partnerships between diverse, often divergent and unfamiliar viewpoints. In terms of the specific components, the complementarity of VA and PAR was highlighted. The combination of international standards with techniques adaptable to local contexts was received positively. VA provides information on levels and causes of mortality in representative surveillance populations but less on the mediating processes and social norms through which exposure to risk originates and accumulates. PAR provides rich data on complex problems embodied in human stories and workable priorities for action, but does not seek to establish generalizability in the same way as statistical methods. The connection to HDSS for timely data representative of district populations was also seen as important to link to timely, robust data and to develop long-term partnerships.

**Table 3:** Key insights and transferrable learning

Key insight	Transferrable learning
Infrastructure for evidence-based decision-making required at operational levels of the health system	Develop neutral research spaces for diverse actors to engage in collaborative evidence production and exchange; Enable research/practitioner/community collaboration to enable relevant research and application; Consider combining quantitative and qualitative data to generate meaningful information with and for stakeholders; Foster local partnerships: HDSS provides robust longitudinal data and stable long-term linkages.
Authentic partnerships and trust are required to embed learning initiatives into routine functions	Promote coherent and sustained commitments for authentic practitioner–researcher relationships; Ensure sufficient time to build partnerships among a range of actors and perspectives; Characterize partnerships and engagements with fairness and respect for divergent viewpoints; Ensure ownership and integration, shared inputs, outputs, processes and adaptation into routine activities.
Practicalities of implementation are important to understand to integrate into routine functions	Pursue sustained, reflective processes over time to learn, adapt and integrate into routine functions Understand whether and how the process is compatible with the goals of different institutions involved; Explicitly recognize and collectively explore the risks of adding administrative burdens; Consider involvement of stakeholders beyond the health system to address social determinants.
Institutional contexts can support (or hinder) local data systems	Recognize that rural PHC is organized and delivered through often highly dynamic and challenging institutional contexts; Facilitate transferability of methods to advance HPSR between research groups and health authorities; Be aware of context, supportive institutional contexts can enable partnerships and processes; Seek support from national and regional research to policy initiatives.

### Building sustainable practice

Adapting the process in an iterative learning fashion and exploring integration into routine functions were seen as potential routes to further develop the method and expand relevance and benefits. The need to negotiate authentic partnerships and develop trust and ownership for this purpose was recognized. While the process was received positively as described above, it was acknowledged that fuller forms of engagement take time and commitment often in challenging and unpredictable environments. Our process—in which researchers and practitioners came together, articulated needs, generated and interpreted data—was seen as a suitable starting point for future collaboration. Building interpersonal and institutional trust is understood as a continuous process characterized by fairness and respect for divergent viewpoints (Cleary *et al.*, 2018b). Researchers developing deliberative processes with stakeholders from diverse institutional settings acknowledge the value of inclusivity, ownership and integration, that can be achieved through enabling shared inputs, outputs, processes and adaptation into routine activities (Nielsen *et al.*, 2017; Rasanathan *et al.*, 2018; Tetui *et al.*, 2018). Ownership needs to exist in an institutional context that supports and recognizes local level data and data processes, however. In this setting, there is evidence of top-down governance cultures that may fail to benefit from innovation and creativity at operational levels (Gilson *et al.*, 2018).

Furthermore, while the platform may be a potentially useful approach and closer collaboration and integration appear intuitively useful, more understanding is needed on practicalities of implementation over time, including whether and how research can inform policy, programming and implementation and in terms of ensuring that processes and outcomes are compatible with the goals and values of the institutions involved (Hamel and Schrecker, 2011; Braithwaite, 2018; Tetui *et al.*, 2018). It is also important to acknowledge that despite the potential to facilitate and enable change, co-production initiatives may also add another administrative process in an already overburdened system. The suggestion that local-level evidence gathering and reflection will enable more effective policy generation, implementation and improved outcomes is thus suggested but remains unproven. As the process develops, it is important to ensure that it is not just another ‘tick-box’ exercise for overloaded staff and managers. Further development inclusive of adaptation, articulation of needs, inputs and skills, and assessment of integration into routine activities will be necessary to understand potential in the approach.

Otherwise, the researchers’ locations informed identification of opportunities for collaborative HPSR. Nationally, the recent SAPRIN (South Africa Population Research Infrastructure Network) initiative consolidates and expands HDSS infrastructure as a national asset to guide policies and programmes (DST, 2016; SAPRIN, 2019). Internationally, the Agincourt HDSS is the founding member of the INDEPTH Network (International Network for the Demographic Evaluation of Populations and Their Health) an umbrella organization of 53 member HDSS sites in 20 low and middle income countries (LMICs). INDEPTH has pioneered the Comprehensive Health and Epidemiological Surveillance System (CHESS) as a new generation of surveillance for health systems and policy, which could serve as an input to further application of the method in the context of HDSS (Sankoh, 2015; INDEPTH, 2019). The methodological transition of VA is also an opportunity to advance learning partnerships between research groups and health authorities (Sankoh and Byass, 2014; D’Ambruoso *et al.*, 2017).

Following completion of the study, we engaged with MDoH, the public and communities in the Agincourt study site, technical agencies such as INDEPTH and the WHO, the international research community and developed new postgraduate training and work-based exchange in HPSR 

(VAPAR Learning Platform, 2016a, 2016b, 2016c, 2019a; HSG, 2018). On this basis, we secured commitment and support from health systems officials, community stakeholders and funders to sustain and extend the learning platform, and launched the VAPAR programme (www.vapar.org) with a Memorandum of Understanding (MOU) signed between DoH and the School of Public Health at the University of the Witwatersrand (VAPAR Learning Platform, 2019b). As the process continues, we will explore integrating into routine activities for collaborative problem-solving, developing and analysing data with those who organize, provide and use services, and working at different levels to understand and enable change.

## Ethical approval

For the VA component, secondary analysis of existing anonymized data was undertaken and approved by the authors’ institutes. For the PAR, informed consent was sought from all participants and all identifiable data were anonymized. Consent was obtained from Institutional review boards at the authors’ institutes.
